# Progress towards early detection services for infants with hearing loss in developing countries

**DOI:** 10.1186/1472-6963-7-14

**Published:** 2007-01-31

**Authors:** Bolajoko O Olusanya, De Wet Swanepoel, Mônica J Chapchap, Salvador Castillo, Hamed Habib, Siti Z Mukari, Norberto V Martinez, Hung-Ching Lin, Bradley McPherson

**Affiliations:** 1 Institute of Child Health and Primary Care, College of Medicine, University of Lagos, Lagos, Nigeria; 2 Department of Communication Pathology, University of Pretoria, Pretoria, South Africa; 3 Hospital Sao Luiz, Sao Paulo, Brazil; 4 Audiology and Phoniatrics Department, México Children's Hospital, 'Federico Gómez" Dr. Márquez 162, Colonia Doctores, 06726 México City, Mexico; 5 Pediatric Department, Faculty of Medicine, King Abdulaziz University, Jeddah, Saudi Arabia; 6 Department of Audiology & Speech Sciences, Faculty of Allied Health Sciences Universiti Kebangsaan Malaysia, Jalan Raja Muda Abdul Aziz, Kuala Lumpur, Malaysia; 7 Better Hearing Institute, Manila, Philippines; 8 Department of Otolaryngology, Hearing and Speech Centre, Mackay Memorial Hospital, Taipei, Taiwan; 9 Division of Speech and Hearing Sciences, Faculty of Education, University of Hong Kong, Hong Kong, China

## Abstract

**Background:**

Early detection of infants with permanent hearing loss through infant hearing screening is recognised and routinely offered as a vital component of early childhood care in developed countries. This article investigates the initiatives and progress towards early detection of infants with hearing loss in developing countries against the backdrop of the dearth of epidemiological data from this region.

**Methods:**

A cross-sectional, descriptive study based on responses to a structured questionnaire eliciting information on the nature and scope of early hearing detection services; strategies for financing services; parental and professional attitudes towards screening; and the performance of screening programmes. Responses were complemented with relevant data from the internet and PubMed/Medline.

**Results:**

Pilot projects using objective screening tests are on-going in a growing number of countries. Screening services are provided at public/private hospitals and/or community health centres and at no charge only in a few countries. Attitudes amongst parents and health care workers are typically positive towards such programmes. Screening efficiency, as measured by referral rate at discharge, was generally found to be lower than desired but several programmes achieved other international benchmarks. Coverage is generally above 90% but poor follow-up rates remain a challenge in some countries. The mean age of diagnosis is usually less than six months, even for community-based programmes.

**Conclusion:**

Lack of adequate resources by many governments may limit rapid nationwide introduction of services for early hearing detection and intervention, but may not deter such services altogether. Parents may be required to pay for services in some settings in line with the existing practice where healthcare services are predominantly financed by out-of-pocket spending rather than public funding. However, governments and their international development partners need to complement current voluntary initiatives through systematic scaling-up of public awareness and requisite manpower development towards sustainable service capacities at all levels of healthcare delivery.

## Background

Chronic and non-communicable diseases have attracted growing attention lately due predominantly to their associated fatality which was estimated as 35 million deaths in 2005 [1]. This figure is twice the number of deaths from all infectious diseases (including HIV/AIDS, tuberculosis and malaria), maternal and perinatal conditions, and nutritional deficiencies combined. Non-communicable diseases are also associated with life-long disabilities which are rarely addressed except through an attempt to describe the associated burden of disease in terms of disability-adjusted life years (DALYs) or similar population health metrics to make them comparable to all other diseases [2]. This approach is an important consideration in the Disease Control Priorities Project for developing countries [3]. While the appropriateness of DALYs in regions with poor data has been debated [4], chronic and life-long conditions of childhood onset such as permanent hearing impairment were excluded from this report which may under-represent its public health significance and current initiatives to address this problem.

Permanent disabling hearing impairment (>40 dBHL) is a significant contributor to the global burden of disease on individuals, families, communities and countries, affecting about 250 million people worldwide as at 2005 [1]. The prevalence of disabling hearing impairment was 120 million in 1995 when the World Health Assembly (WHA) passed a resolution on the Prevention of Hearing Impairment urging member states to *"prepare national plans for the prevention and control of major causes of avoidable hearing loss, and for early detection in babies, toddlers and children, as well as in the elderly, within the framework of primary health care" *[5]. This estimate has more than doubled in a decade with two-thirds of those with hearing impairment living in developing countries and about 25% are of early childhood onset [1]. Permanent hearing impairment is an aetiologically heterogeneous trait attributable to genetic and environmental causes. Among the environmental causes are infectious diseases that account for substantial infant mortality in developing countries and which are currently addressed through various global health programmes by UN agencies and their development partners [6,7]. However, a significant proportion of hearing impairment is not preventable. Moreover, the exclusion of childhood hearing impairment in current global health priorities may further delay the implementation of the WHA resolution in many countries where national health policies are tailored to reflect on-going global health initiatives which unfortunately are not yet geared towards early childhood development.

Of all sensory disabilities in early childhood permanent hearing impairment that originates from birth or in the neonatal period is of special interest because of its adverse consequences on speech, language, cognitive and psychosocial development and the subsequent impact on educational and vocational attainment when detected late particularly in developing countries [8]. Detection and intervention within the first year of life is crucial for and often associated with favourable developmental outcomes [9-11]. Primary prevention through immunisation, health education, improved maternal and child health services are useful for addressing environmental causes but limited in dealing with the full spectrum of neonatal hearing loss attributable to genetic or hereditary aetiologies [6,7]. Screening of newborns or infants before the age of three months has emerged within the last decade as an effective secondary prevention strategy for the early detection of disabling permanent hearing impairment. The development and availability of objective hearing tests using otoacoustic emissions (OAE) and/or auditory brainstem response (ABR) which are suitable for testing babies from birth has been a major impetus for this trend. These screening tests are often automated, reliable, non-invasive and simple-to-use by primary healthcare workers. Although universal or selective screening of newborns is widely implemented in developed countries, its introduction in the developing world may be constrained by reservations concerning the necessity of such a programme because of prevailing adverse health and socio-economic conditions. For instance, the general principles for screening programmes first stated by Wilson and Jungner in 1968 [12] are often misinterpreted to suggest that an infant hearing screening programme should not be implemented until adequate follow-up services are available to cater for children detected with hearing loss [13,14]. However,  it is difficult to justify such a requirement for prelingual hearing loss which will inevitably become manifest even without screening and for which optimum intervention is time-bound  [9-11]. In fact, it is not uncommon in traditional communities for parents to resort to unorthodox and harmful therapeutic treatments for the unusual behavioural manifestations of hearing loss during the period of uncertainty [15-17].

The healthcare systems in many developing countries are fragile and government funding is uncertain due to competing demands from diseases with high mortality rates such as tuberculosis, HIV/AIDS and malaria. However, there is currently no intervention for any disease or health condition in the developing world with adequate services to meet the requirements for all those in need. This has led to the concept of "scaling up" which requires the systematic development and expansion of intervention services to progressively address established healthcare needs [18-20]. Ideally, health needs are best established through sound population-based epidemiological data. However, such data are often lacking and difficult to obtain especially for non-life threatening conditions in developing countries [21-23]. Rather, pilot studies have been valuable for demonstrating the feasibility of planned programmes, establishing service needs and for identifying potential challenges [24]. This strategy has also been successfully applied for the introduction of universal newborn hearing screening in many developed countries [25-28].

Recently, Morton & Nance aptly observed that newborn hearing screening is "a silent (global) revolution" which is "an achievable and important goal for all nations" [29]. Reports are already emerging from a growing number of developing countries that have voluntarily implemented pilot programmes or projects on early hearing detection [30], but lessons from these efforts have not yet been documented to provide relevant baseline data to facilitate global initiatives towards systematic capacity-building for requisite services. This cross-country study therefore set out to examine the progress achieved so far in developing countries in relation to current screening strategies, the funding mechanism for screening services, disposition of parents and healthcare professionals towards such programmes and areas where improvements are most indicated.

## Methods

### Study design

This study was designed as a cross-sectional, descriptive and questionnaire-based survey complemented by other relevant reports on infant hearing loss in developing countries in view of the wide variations in practices and the lack of a standardised screening protocol across countries.

### Survey instrument and themes

A structured questionnaire was designed to ascertain the following information from each country surveyed [see Additional file 1]:

• *Type(s) of infant hearing screening models implemented (if any)*

• *Financing mechanism for screening services*

• *Parental and health professionals' attitudes towards infant hearing screening*

• *Achievements and challenges of infant hearing screening programmes*

The questionnaire did not require the respondents' active involvement in an infant hearing screening project but their familiarity with the subject matter in their respective countries.

### Data collection

The survey instrument was sent via e-mail to a cross-section of researchers and ear care specialists (physicians, ENT surgeons and audiologists) resident in each of the sub-regions that make up the developing world based on UNICEF/World Bank classification namely: Sub-Saharan Africa [SSA], Middle East/North Africa [MEN], South Asia [SOA], East Asia/Pacific [EAP] and Latin America/Caribbean [LAC], excluding Central/Eastern Europe [CEE]. Taiwan and Hong Kong were considered independently of mainland China to highlight the historical antecedents to infant hearing screening in these locations.

Prospective respondents were identified from published work in PubMed or in the public domain relating to hearing impairment in developing countries. Those contacted were informed of the purpose of the survey, which was to gather up-to-date and representative information on activities relating to early hearing detection and intervention in the developing world. The target was to enlist at least two countries to represent each sub-region. In order to make up for the lack of response from a particular region, we relied on published articles from the region. Moreover, responses obtained from the survey were supplemented with information from relevant articles in Medline/PubMed (1996 – 2006), abstracts of presentations at the biennial international conference on newborn hearing screening NHS2006 at Lake Como, Italy [30] and an electronic search of the internet. The PubMed search was backdated to 1996 to ascertain the progress after the 1995 WHA resolution. The NHS2006 was selected because this biennial meeting is perhaps the largest and the only international conference wholly dedicated to newborn hearing screening outside the USA.

### Criteria for evaluating performance of screening programmes

The conventional criteria for evaluating the performance of an infant hearing screening programme in developed countries are often related to the following JCIH (2000) benchmarks [31]:

• *Screen should be offered to a minimum of 95% of eligible infants before hospital discharge or within one month of age [Screening Coverage]*

• *The referral rate following the screening process should not exceed 4% [Screening Efficiency].*

• *The return-for-follow-up rate should not be less than 95% of infants who failed the screening tests and were referred for diagnostic evaluation [Return Rate]*

• *Diagnostic evaluation is provided for infants who failed the screening tests before 3 months of age and enrolled in a family-centred early intervention programme before 6 months of age [Age of Diagnosis and Intervention].*

There are presently no benchmarks for evaluating infant hearing screening programmes in developing countries. What constitutes "reasonable standards" would be expected to reflect the realities and scope of possibilities in this region. Nonetheless, valuable insights can still be ascertained from evaluating the performance of pilot screening programmes that have been documented based on the JCIH criteria.

### Data analysis

The emphasis in this descriptive study was on qualitative analysis of available information from the questionnaire and internet searches. The quantitative data relating to the performance of screening programmes were obtained from peer-reviewed publications (PubMed) or from abstracts of presentations at the NHS2006 conference, which may take up to 2 years before they become available in PubMed when published. For ease of analysis, the responses to the questionnaire on the attitudes of parents and professionals towards newborn hearing screening were structured on a Likert scale and scored as follows: very negative (1), negative (2), not sure (3), positive (4), and very positive (5).

## Results

Sixteen (88.9%) of the 18 professionals contacted in Kenya, Nigeria, South Africa [SSA], Oman, Saudi Arabia, Iran [MEN], India, Pakistan, Nepal, Bangladesh [SOA], China, Hong Kong, Taiwan, Malaysia, Philippines [EAP], Brazil, Mexico and Chile [LAC] responded. They comprised two paediatricians, nine otolaryngologists and five audiologists - typically reflecting the current multidisciplinary intervention for infant hearing loss globally. No responses were received from Kenya and Pakistan at the time of this report. Nepal and Bangladesh were excluded in the final analysis as the respondents reported that no infant hearing screening programme had commenced in these countries at the time of the survey despite substantial presence of multilateral institutions and donor agencies. Three more countries (Jordan, Qatar and Singapore) and Pakistan were added to the list of countries based on the results from our electronic searches of the internet and PubMed making a total of 18 countries including Hong Kong and Taiwan as shown in Table 1[32-49].

**Table 1:** Demographic characteristics of countries with infant hearing screening programmes

**REGION/COUNTRY**	**Per Capita Income (US$)**	**Total Population ('000)**	**Under 5 Population ('000)**	**Annual Live Births ('000)**	**Born in Hospitals (%)**	**Born Outside Hospitals (%)**	**Infant Mortality ('000)**	**Life Expectancy (Years)**	**Total Health Expenditure (2003)**	
									
									**% Public**	**% Private**
**Sub Sahara Africa [SSA]**										
Nigeria	390	128,709	21,943	5,323	35	65	101	43	26	74
South Africa	3,630	47,208	5,248	1,093	84	16	54	47	39	61
**South of Asia [SOA]**										
India	620	1,087,127	120,155	26,000	43	57	62	64	25	75
Pakistan	600	154,794	20,922	4,729	24	76	80	63	28	72
**Middle East [MEN]**										
Saudi Arabia	10,430	23,950	3,178	665	91	9	21	72	76	24
Iran	2,300	68,803	5,890	972	90	10	32	71	47	53
Qatar	12,000	777	85	14	99	1	18	73	74	26
Jordan	2,140	5,561	734	150	100	0	23	72	45	55
Oman	7,830	2,534	302	64	95	5	10	74	83	17
**East Asia Pacific [EAP]**										
China	1,290	1,307,989	86,055	17,372	96	4	26	72	36	64
Malaysia	4,650	24,894	2,38	549	97	3	10	73	58	42
Philippines	1,170	81,617	9,873	2,028	60	40	26	71	44	56
Singapore	24,220	4,273	226	40	100	0	3	79	36	64
**Latin America [LAC]**										
Brazil	3,090	183,913	17,946	3,728	96	4	34	71	45	55
Mexico	6,770	105,699	10,982	2,201	95	5	23	75	46	54
Chile	4,910	16,124	1,246	249	100	0	8	78	49	51

Sources: UNICEF State of the World's Children 2006; WHO World Health Report 2006

### Demography of countries with infant hearing screening programmes

**Figure 1:**
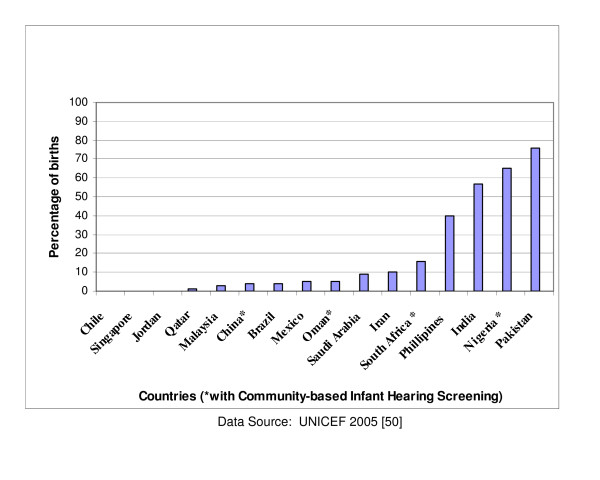
Proportion of births outside hospital facilities.

**Figure 2:**
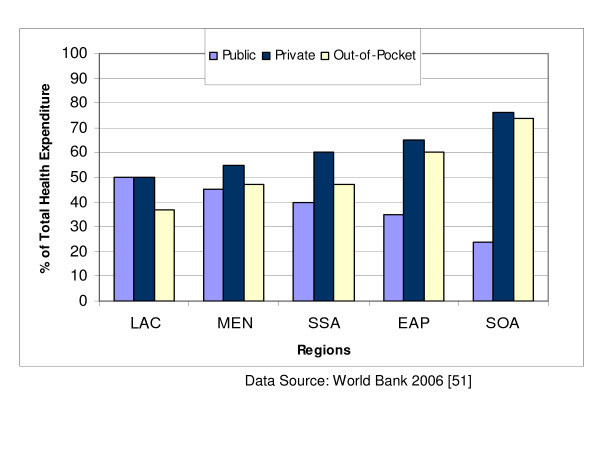
Health expenditure patterns in developing regions. Latin America & the Caribbean **[LAC]**, Middle East & North Africa **[MEN]**, Sub-Saharan Africa **[SSA]**, East Asia & Pacific **[EAP]**, South of Asia **[SOA]**

**Figure 3:**
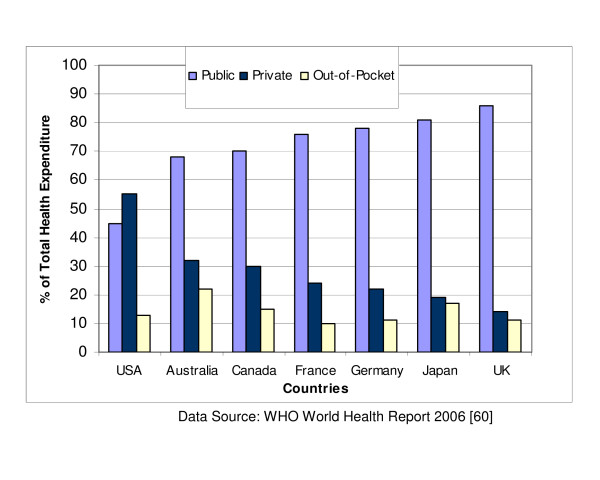
Health expenditure patterns in developed countries.

The demographic characteristics of these countries are presented in Table 1, excluding those for Hong Kong and Taiwan which are currently not provided by any of the UN agencies [50]. The per capita income varies from US$390 in Nigeria to US$24,220 in Singapore. The World Bank classifies countries with national income per capita less than US$826 as low income; US$826 – US$10,065 as middle income; and above US$10,065 as high income. On this basis, Saudi Arabia, Qatar and Singapore are high income developing countries with a good prospect of having a standard of living and healthcare comparable to levels in many developed countries. China and India are the most populous countries and also have the highest annual live births of 17.4 million and 26 million respectively. In contrast, the annual live births are as low as 14,000 in Qatar, 40,000 in Singapore and 64,000 in Oman. The majority of births in Nigeria, India and Pakistan occur outside regular hospital facilities (indexed by the proportion of skilled attendants at birth) as displayed in Figure 1. Infant mortality rate ranges from less than 20 per 1000 live births in Qatar, Oman, Malaysia, Singapore and Chile to over 60 per 1000 live births in Nigeria, India and Pakistan while life expectancy is lowest in Nigeria (43 years) and South Africa (47 years) compared with 79 years in Singapore or 78 years in Chile. Private spending on healthcare accounts for over half of national health expenditure in all countries except Saudi Arabia, Qatar, Oman and Malaysia. Government contributions to total expenditure for healthcare services do not exceed 50% in all the regions of the developing world (Figure 2) [51]. Private spending accounts for as high as 75% of total health expenditure in South Asia and not less than 60% in Sub-Saharan Africa in sharp contrast to the pattern in developed countries (except USA) where public expenditure accounts for over 70% of total health expenditure (Figure 3). Private spending could be through private health insurance, employer funding or out-of-pocket expenditure. Out-of-pocket spending is the direct consumer financial contribution to health care by individuals after deducting health insurance, employer funding and other private payments for health care. It accounts for the bulk of private health expenditure in all regions of the developing world.

### Profile of infant hearing screening projects

The earliest reported project using electrophysiological tests of all the 18 countries identified was perhaps initiated in India in 1986 among high risk infants, based on the responses to the questionnaire survey (Table 2). As with all the countries surveyed, services are concentrated in urban areas presumably because of accessibility to available audiological services. The second oldest screening programme is in Brazil and dates back to 1988. There are over 237 screening sites across many states in this country and the programme is by far the largest in any developing country. In Oman universal newborn hearing screening is currently offered routinely nationwide after prior pilot studies in various regions of the country. This is perhaps the first developing country with a national programme on newborn hearing screening. Chile has just implemented a national initiative to screen almost half of the babies born in the country based on known risk factors for hearing loss. In Iran, pilot studies have also been conducted in 28 of its 30 provinces [36].

**Table 2:** Profile of infant screening programmes in developing countries

**REGION/Country [Related reference(s)]**	**Data Source(s)**	**Year; (Location)**	**Model**	**Protocol**	**Government Funding**	**Patient Obligation**	**Support Programme**
*Sub Sahara Africa*							
**Nigeria **[32]	Survey, PubMed	2005 (Lagos)	Hospital, MCC (UNHS)	OAE AABR	None	None	None
**South Africa **[33]	Survey, PubMed	2003 (Pretoria)	Hospital, MCC: TNHS	OAE	None	None	None
*South of Asia*							
**India **[]	Survey, Internet NHS2006	1986 (Mysore, Kochi, Chennai)	Hospital: TNHS	OAE	Screening	Diagnosis; Hearing aids	Pre School & School Entry
**Pakistan **[34]	Internet	1999 (Lahore)	Hospital: UNHS	OAE	N/A	N/A	N/A
*Middle East*							
**Saudi Arabia **[35]	Survey, PubMed	1996 (Jeddah)	Hospital: UNHS	OAE	None	All Services	School Entry
**Iran **[36]	Survey, NHS 2006	2002 (Tehran, Mashad)	Hospital: UNHS	OAE	None	All services	Pre School
**Qatar **[37]	NHS2006 PubMed	2003 (Doha)	Hospital: UNHS	OAE	Screening, Diagnosis	N/A	N/A
**Jordan **[38]	PubMed	2001 (Multiple Cities)	Hospital, MCC: UNHS	OAE	Minimal	N/A	N/A
**Oman **[39]	Survey, PubMed	2001 (Multiple cities)	Hospital, MCC: UNHS	OAE, AABR	Screening, Diagnosis	None	School Entry
*East Asia Pacific*							
**China **[40]	Survey, NHS2006 PubMed	1999 (Shanghai, Beijing, Shandong)	Hospital MCC : TNHS UNHS	OAE AABR	Education Materials	Screening Diagnosis	Pre-School
**Hong Kong **[41]	Survey, PubMed	1998 (Hong Kong	Hospital, MCC TNHS, UNHS	OAE AABR	Screening	Diagnosis; Hearing aid	Pre-School & Sch Entry
**Taiwan **[42,43]	Survey, PubMed	1998 (Taipei)	Hospital: UNHS	OAE, AABR	No	All Services	Pre-School
**Malaysia **[44]	Survey, PubMed	2000 (Kuala Lumpur)	Hospital: UNHS	OAE	Screening, Diagnosis	Hearing aids	Pre-School & Sch Entry
**Philippines **[45]	Survey PubMed	2000 (Manila)	Hospital: UNHS	OAE	None	All services	None
**Singapore **[46,47]	PubMed NHS2006	2002 (Singapore)	Hospital: UNHS	AABR OAE	None*	All services	N/A
*Latin America *							
**Brazil **[48]	Survey, PubMed	1998 (Multi cities)	Hospital: TNHS, UNHS	OAE	No	All Services	Pre-School & School Entry
**Mexico **[49]	Survey, PubMed	2005 (MexicoCity)	Hospital: TNHS, UNHS	OAE AABR	Screening	Diagnosis; Hearing aid	School Entry
**Chile **[]	Survey, Internet	2005 (National)	Hospital TNHS	OAE AABR	Full	None	School Entry

N/A: Not Available *After the initial pilot studies; OAE: Oto-acoustic emissions; AABR: Automated Auditory Brainstem Response MCC: Maternal & Child Health Centre; TNHS: Targeted Newborn Hearing Screening; UNHS: Universal Newborn Hearing Screening

Overall, screening protocols consist of both universal and targeted approaches in which OAE and/or AABR techniques are employed. The targeted screening is commonly based on the risk factors recommended by the JCIH [31]. The majority of screening programmes are hospital-based except in Nigeria, South Africa, Taiwan and Hong Kong (and to a limited extent in China, Jordan and Oman) where community-based programmes have been reported. Community-based programmes are typically implemented during routine immunisation clinics at community health centres [32,33,41].

Existing healthcare personnel in hospital-based projects are more commonly entrusted with screening except in a few countries like Nigeria where non-specialists are recruited and specially trained to conduct screening. Screening in hospitals is predominantly handled by nurses, audiologists or otolaryngologists but diagnostic tests are conducted by audiologists or otolaryngologists. Community-based programmes are more commonly handled by non-specialists who have received training in the use of screening instruments. However, the screening staff rely on the existing primary healthcare workers in educating parents on the value of the programme.

### Funding strategies for infant hearing screening services

Most of the pilot projects were privately initiated by researchers and services were offered in public and private hospitals. For programmes implemented in public hospitals government funding, if available, was usually limited to providing free screening or for public awareness campaigns/materials (Table 2). Data from most of the middle-to-high income countries were scanty and it is most likely that public funding in many of these countries may extend to free diagnostic tests and provision of hearing aids. For instance, a 4-year pilot UNHS programme in Singapore and the emerging national programmes in Chile and Oman are also publicly funded [39,46]. In Nigeria, the UNHS pilot studies at five locations have been funded through a combination of public and private sources including donation or loan of equipment by manufacturers. The programme in Nigeria is currently offered at no charge to parents up to the provision of hearing aids through funding support from a local non-governmental organisation. In Brazil, private hospitals may charge between US$25 and US$40 to screen a baby. The cost of screening a baby was generally not reported in most studies and would normally be expected to depend on several factors like the type of equipment used, the model of screening, the level and calibre of personnel conducting the programme and other overhead expenses. In Oman, the cost of screening a baby was estimated as US$7.10. There was no evidence that any of the countries in this review had a national health insurance scheme incorporating hearing screening services, which may indicate that private contributions to health expenditure were predominantly derived from out-of-pocket spending by individual health seekers. In addition, it was difficult to establish the cost of post-screening services such as diagnostic tests and fitting of hearing aids. The costs of cochlear implants were reported to vary from US$25,000 to US$30,000 in South Africa but similar information was not readily available in countries such as Brazil, Mexico, Iran, India and China where they are also currently offered.

### Parental and healthcare professionals' attitudes towards infant hearing screening

**Figure 4:**
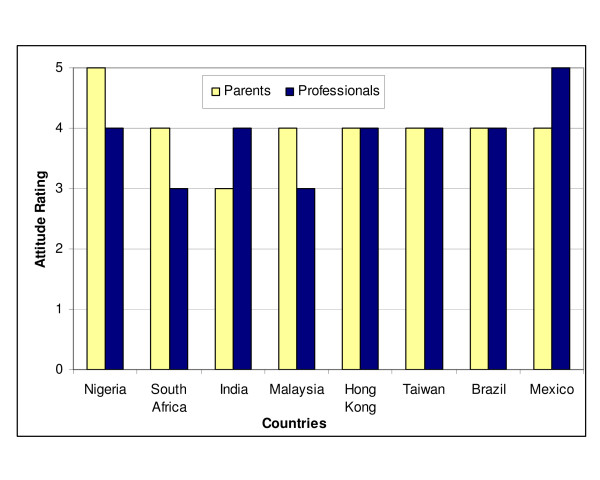
Attitudes of parents and health professionals towards infant hearing **screening **[Key: Very negative (1); Negative (2); Not sure (3); Positive (4); Very positive (5)]

Parental attitude was reported to be generally positive based on the responses to the survey, except in India where it was rated as uncertain (Figure 4). However, parental opposition has not been reported in the few infant hearing screening projects that have been undertaken so far in India or in any other developing country. Parental attitude was rated as most positive in Nigeria. Similarly, the attitudes of health professionals were mostly rated as positive except in South Africa and Malaysia where some ambivalence has been noted. Professional support appears to be most enthusiastic in Mexico. The attitudes of parents and health professionals were rated equally in Hong Kong, Taiwan, Malaysia, Saudi Arabia and Brazil. It is pertinent to observe that these attitudinal ratings are principally based on anecdotal evidence rather than systematic investigation of parents and professionals. Only very limited published data exist on the current knowledge and attitude of health professionals towards hearing screening in developing countries. Evidence from the limited information obtained from parents who have participated in screening programmes or parents of hearing impaired children generally indicate that infant hearing screening is highly desirable [52,53]. One UNICEF-supported study in Iran intended to establish the knowledge, attitudes and practices of parents of children with various disabilities found that 73% of parents of deaf children had a favourable attitude and 72% had "correct knowledge" [54]. However, another study among families of babies 4 months or less attending immunisation clinics in Singapore reported that 59% of those whose babies had missed hearing screening at birth refused the offer of screening at the immunisation clinics because they doubted their babies had a hearing loss, or that they felt parents can monitor the babies hearing themselves or that they needed to wait till the baby is older [46].

### Performance of infant hearing screening programmes

Table 3 shows the available published data from a cross-section of countries. Pilot studies based on target screening are not considered because of the lack of uniform performance criteria for such programmes.

**Table 3:** Performance of universal infant screening pilot programmes in developing countries

**Region/Country [Reference]**	**No of Births or Eligible Infants**	**Total Screened (% Coverage)**	**Referral at Discharge (%)**	**Returned for Follow-up (%)**	**Referred for Diagnosis**	**Returned for Diagnosis (%)**	**Mean Age of Diagnosis (months)**
*Sub Saharan Africa*							
**Nigeria **[32]	1,132	1,132 (100)	204 (18.0)	116 (56.9)	57	32 (56.0)*	2.0*
**South Africa **[33]	510	489 (95.9)	68 (13.9)	27 (39.7)	9	1 (11.1)	6.0*
*South of Asia*							
**Pakistan **[34]	n/a	756	77 (10.2)	26 (33.8)	6	6 (100)	6.0
*Middle East*							
**Saudi Arabia **[35]	13,071	11,986 (91.7)	300 (2.5)	300 (100)	22	22(100)	5.5
**Oman **[39]	32, 080	21,387 (66.7)	262 (1.2)	262 (100)	55	36 (65.5)	n/a
*East Asia Pacific*							
**Hong Kong **[52]	1,076	1,064 (98.9)	476 (44.7)	n/a	37	35 (94.6)	n/a
**Taiwan **[42]	7 7,496	6,765 (90.2)	430 (6.4)	370 (86.0)	154	140 (90.9)	3.5
**Malaysia **[44]	5,242	4,437 (84.6)	531 (12.0)	303 (57.0)	55	34 (61.8)	3.6
**Singapore **[47]	44, 579	44,488 (99.8)	1040 (2.3)	940 (90.4)	254	194 (76.4)	2.7
*Latin America*							
**Brazil **[48]	4,631	4,196 (90.6)	73 (1.7)	60 (82.2)	25	25 (100)	4.3
**Mexico **[49]	3,069	3,066 (99.9)	33 (1.1)	32 (97.0)	7	7 (100)	3.0

*unpublished

n/a not available

The lowest screening coverage of 66.7% was reported in Oman and is largely attributable to the inception of a nation-wide screening programme rather than a pilot study. The recommended target of 95% coverage was achieved in Nigeria, South Africa, Hong Kong, Singapore and Mexico. Singapore screened nearly all of its 44,579 eligible babies which was the highest number of babies screened of all countries considered in this review. The referral rate at discharge was lowest in Oman at 1.2% and highest in one Hong Kong pilot study, at 44.7%. The latter is attributable to the pilot screening protocol which required all babies to undergo three stages of test with OAE [52]. The referral rates in Saudi Arabia, Oman and Brazil fell within the recommended target of 4% or less. The "referral rate prior to discharge" was preferred to eliminate the differences in the number of repeat tests across various protocols. The rate of "returned-for-follow-up" after discharge from the hospital or the screening centre is an index of how effective the tracking system is as well as the voluntary disposition towards the completion of the screening by parents or the logistics of returning to the screening centres. The lowest rate of 39.7% was recorded in South Africa where the screening programme was implemented during routine visits for immunisation. The recommended target of a minimum of 95% was only attained in Saudi Arabia and Oman. The rates for Taiwan (86.0%) and Brazil (82.2%) are impressive for initial stages of their programmes. Information on the age of diagnosis and enrolment in family-centred intervention services were rarely reported. Where available, age of diagnosis varied from 2.7 months in Singapore to 5.5 months in Saudi Arabia [35,46]. In the absence of UNHS, age of diagnosis varies from about 18 to 86 months [55,56].

## Discussion

In an earlier report on the feasibility of infant hearing screening in developing countries a number of challenges were identified that may impede the implementation of such a programme [57]. But it was equally noted that these challenges were not insurmountable given the variety of options available at different levels of healthcare delivery. Notwithstanding the myriad constraints in developing countries, our current report demonstrates that early hearing detection programmes are feasible as a public health initiative and are required to facilitate the systematic development of requisite services and the implementation of WHA 48.9 resolution [5].

### Screening models and platforms

The screening models that have been implemented so far range from the most basic of targeting high risk infants for screening to universal screening with OAE and/or AABR. Cost considerations significantly influence the choice of screening protocol as do the logistics of achieving effective coverage and screening performance. The use of non-specialists as screeners at primary healthcare level has been found to be cost-effective while regular health workers are effective in educating parents on the programme. Restricting screening to highly skilled personnel like audiologists or other ear care specialists may not serve the course of a rapid spread of infant hearing screening as an important public health programme due to the general dearth of such manpower. Experiences from countries like Nigeria and India also suggest that depending on primary care physicians to refer high-risk babies to a central location for screening is ineffective. Hospital-based screening programmes are essential in all countries but it is also necessary to have complementary community-based programmes especially in countries where a significant proportion of births occur outside hospitals. Most community-based programmes are linked to visits to maternal and child clinics for routine immunisation in the first three months of life. In India, incorporating infant hearing screening into the country's Integrated Child Development Services (ICDS) has been considered as a feasible option. The ICDS is reputed as the world's largest community-based outreach programme with over 40,000 centres nationwide [58]. It reaches more than 34 million children aged 0–6 years and 7 million pregnant and nursing mothers. However, screening babies older than 3 months in community-based programmes could be difficult as they may be too restless to be tested with OAE or AABR without sedation. Moreover, finding suitable test environments especially in busy hospitals or community health centres may also present some challenges that must be managed creatively across potential locations. Tolerance limits of screening equipment for ambient noise, durability of consumables, access to timely technical support and availability of loanable equipment when instruments are sent for maintenance/repairs should not be overlooked in the choice of equipment manufacturers. Although cost-effectiveness analysis data are not currently available from developing countries, it may be useful to consider targeted screening as a possible take-off point where cost is a major hurdle. For instance, prevailing adverse perinatal events that account for high infant mortality in some countries have been associated with permanent hearing impairment [7]. Since some of these factors can be readily ascertained even from parents they may serve as useful criteria for initiating community-based targeted screening. However, pilot programmes based on universal infant hearing screening provide more useful information for planning purposes and for establishing vital epidemiological data like the incidence of and/or risk factors for hearing impairment more accurately.

### Funding issues

The costs of acquiring and maintaining screening equipment at community levels could be a major concern for the rapid expansion of programmes. Typical OAE screeners cost about US$3,000 and AABR about US$8,000 excluding consumables and replacement parts. To ensure that screening is uninterrupted at least two of such screeners would be required even at the least busy birthing centres and many more would be required if the screening protocol combines both OAE and AABR.

Most of the concerns often expressed in relation to funding arise from misconceptions about the current role of government in healthcare financing in developing countries [59]. Government contributions to national health expenditure in developed countries range from an average of 80% in Europe, 81% in Japan, 70% in Canada, 68% in Australia to 45% in USA [60]. This contrasts sharply with the levels in developing countries where government contributions rarely exceed 50% and are as low as 25% in India and 26% in Nigeria. Although private health expenditure accounts for 55% of the total expenditure in USA, only 24% of this amount is attributed to out-of-pocket spending – in sharp contrast to India or Nigeria where out-of-pocket spending accounts for at least 90% of private health expenditure. In effect, out-of-pocket spending accounts for about 13% of expenditure in USA or 10% in Europe compared with 75% in India or 76% in Nigeria. These data would suggest that it may be unethical to predicate the decision for introducing new health interventions in most developing countries solely on government financial capacity if the consumers ultimately will pay for the services. The way individuals choose to order their spending priorities is often unpredictable and complex. For instance, some communities may consider socially stigmatised diseases more important than non-stigmatised diseases even if they are less prevalent [61]. The scarcity of public funding in some of the countries perceived as poorly-resourced should therefore not constitute a deterrent to the systematic development of infant hearing screening services in these countries.

Governments in low and middle income countries are unlikely to ever have adequate resources to cater for all the myriad healthcare needs of their citizens. It is more plausible to see the role of government as facilitators of public-private partnerships in which its preoccupation will be towards creating public awareness and setting standards for best practices rather than the direct provision of services especially for non-fatal chronic conditions. This role should also extend to ensuring that training curricula of health professionals provide up-to-date skills for the broad spectrum of prevailing healthcare needs. Multilateral institutions such as the World Bank, UNICEF, WHO and their funding partners can play a valuable role in providing financial and relevant technical expertise to support pilot studies. Such studies have been known to be successful if they are preceded by the inclusion of goal(s) for early hearing detection and intervention in the national health policy [32,62]. All these initiatives will have a good chance of success if they are implemented within the stepwise framework recently articulated by WHO [63]. It is also pertinent to acknowledge current efforts by WHO and other organisations towards the provision of guidelines for audiological services as well as the development of low-cost solar powered hearing aids costing no more than US$40 per piece compared to about US$500 dollars in developed countries [64]. Collaborative initiatives at the global level between multilateral institutions, their donor partners and manufacturers towards making screening equipment and the associated costs more affordable would also be desirable. Having one diagnostic centre in a district/province serving several hospitals that only conduct screening as has been successfully implemented in China may prove to be cost-effective [65]. But this approach would have to be balanced with the risk of poor follow-up rate in some other countries. Of the 2 to 6 per 1000 babies with neonatal hearing loss in developing countries [8], those with severe-to-profound hearing loss that require hearing devices may not exceed 2 per 1000. Many communities can still mobilise resources to provide support for those in need as the devices become cheaper through concerted global initiatives. Notable charitable organisations like Christoffel-Blidenmission, Lions Club and Rotary International already have networks for supporting individuals with hearing impairment in many developing countries which can also be channelled towards early hearing detection services.

### Parental and professional support

The attitude of parents also has as much impact in optimising the uptake of screening services as the support of health professionals like nurses and physicians. Initial enthusiasm for screening before hospital discharge may be short-lived due to poor parental education on the value of screening resulting in poor follow-up compliance. Sustaining physician support in environments overwhelmingly oriented towards treating fatal diseases is a major challenge but can be mitigated by government support through on-going public awareness campaigns on the value and efficacy of early detection and intervention [66,67]. Experiences from developed countries would suggest that there are no quick-fixes especially at the early stages of infant hearing screening programmes. Success stories for early intervention are still quite limited while the role of family involvement in the habilitation process is not yet widely appreciated. Another crucial area of support required from government relates to cultural reorientation in communities where current beliefs and attitudes are detrimental to effective parental acceptance of infant hearing screening [15-17,68].

Parents must be sufficiently educated on the benefits and risks of the screening process and their consent subsequently sought prior to conducting the test(s). Every pregnant mother expects and longs to give birth to a healthy baby and to leave the birthing centre with the assurance that their apparently well child has every chance for optimal development. Parental consent for screening can therefore be readily obtained if it is presented as part of routine neonatal examinations after delivery and because the screening procedure itself is painless, non-invasive and quick to administer. However, the health professional must handle the screening process with sensitivity because the arrival of a newborn is an event that is both joyous and emotion-laden for the parents. The most opportune time to begin discussion with parents is probably during ante-natal clinics or sometime before delivery.

Balancing the benefits of screening against its potential harms may leave health professionals with an ethical dilemma because no screening test is perfect. The risks of false assurance that a child has no hearing loss or of unnecessary anxiety for a child falsely detected with hearing loss when in fact the opposite is the case must not be ignored. Practical ways of minimising these risks include delaying screening beyond 48 hours after birth to eliminate the effect of vernix plugs, conducting a two-stage screening with OAE and AABR and minimising the effect of ambient noise during screening, which could be quite a daunting challenge in busy neighbourhoods.

### Programme performance

The impressive coverage from all the screening projects is a strong indication of the high parental acceptance and encouraging support from health professionals. This is not to suggest that difficulties are not occasionally encountered in the course of securing optimum screening up-take. For instance, in Malaysia, the relatively lower coverage was attributed to the challenge of getting nurses involved with screening to effectively combine this function with their other regular duties which are often regarded as more important. In other countries, babies may be discharged before screening because parents are reluctant to wait or the screening facilities cannot cope with the number of babies eligible for screening before discharge. In such circumstances it has been found useful to encourage parents to ensure that their babies are tested during post-natal visits for immunisation to the hospital or community health centres.

High referral rates are common in the initial stages of the screening programme and they improve as the screening teams become more experienced. However, some screening protocols are readily associated with high referral rates especially when screening is limited to only OAE even with multiple testing. AABR instruments may not be affordable and this may account for its exclusion in some locations. Referral rates are usually minimal when OAE and AABR are combined. Service providers would need to make their own judgment on the most desirable trade-off between cost and efficiency especially where certain categories of hearing impairment like auditory neuropathy may be missed in screening programmes solely based on OAE.

Ensuring that parents of babies who failed the screening test prior to discharge return for subsequent follow-up appointments is a major problem in most countries. During the initial screening maternal consent may be occasioned by mothers simply not wanting to feel left-out since the majority of mothers are likely to consent. Return for follow-up may therefore serve as a more accurate index of parental commitment than the coverage achieved for the initial screening. Factors such as transportation costs, parental convenience and anxiety may contribute to a high default rate for follow-up. Parents of children with severe-to-profound hearing loss are sometimes more cooperative when requested to attend follow-up appointments if their babies were tested later than 3 months of age as they may have already begun to suspect the child's hearing difficulty. On some occasions poor return rates are associated with the lack of an effective tracking system or poor communication between health professionals and the parents. These difficulties have also been reported in developed countries and programmes have demonstrated increasing efficiency after implementing improved tracking systems and increasing awareness of hearing loss amongst healthcare professionals and families [69]. Where cost is a major deterrent, it may be useful to consider possible ways of supporting parents such as waiving charges for follow-up services including diagnostic evaluations since the number of babies are likely to be low at this stage in a well-conducted programme.

The limitations that may be associated with this study include the fact that the methodology may not fully represent the current status of early hearing detection and intervention in some countries due to the lack of recent data in the public domain. Similarly, many countries that have initiated infant screening projects may have been omitted. In addition, relying on a single respondent to the questionnaire-survey for each country could have limited the coverage especially in very large countries like India and Brazil. Valuable information for some Latin American countries like Chile or a populous country like China may also not be published in English which restrict their usage for our study. However, the insights from this progress report should serve as baseline data for a more extensive future audit. The lack of published data in many countries is not in itself an evidence of a lack of progress but an indication of the prevailing challenges to the conduct and reporting of epidemiological research in the developing world.

## Conclusion

This report demonstrates that infant hearing screening is a feasible and viable early hearing detection strategy in developing countries. The nationwide implementation of such programmes may be hampered in many countries by low public awareness, resource constraints and lack of government and donor support. However, it is worth noting that no public health intervention is without challenges and those related to infant hearing screening may be daunting but not insurmountable. The most successful public health programmes often start from small beginnings and are followed by systematic scaling up. Universal newborn hearing screening has been mandated in many developed countries not because there were adequate support services in all communities or full support from all health professionals at inception but rather in recognition of the overriding value of early detection and the incentives it provides for the systematic scaling up of services to meet emerging and growing needs. Evidence from a rapidly expanding number of countries such as Brazil, Oman and Chile that have progressed from rudimentary pilot projects to multi-city programmes lend credence to infant hearing screening as an important and achievable public health initiative in the developing world. So also are the many pilot projects that have been sustained since their inception without public funding. These voluntary initiatives are valuable in providing essential data for the planning and systematic development of relevant services towards the effective delivery of an important public healthcare service in developing countries.

## List of abbreviations

**AABR **– Automated Auditory Brainstem Response

**ABR **– Auditory Brainstem Response

**DALYs **– Disability-Adjusted Life Years

**EAP **– East Asia/Pacific

**JCIH **– Joint Committee on Infant Hearing

**LAC **– Latin America/Caribbean

**MCC **– Maternal and Child Centre

**MEN **– Middle East/North Africa

**OAE **– Otoacoustic Emissions

**SOA **– South Asia

**SSA **– Sub-Saharan Africa

**TNHS **– Targeted Newborn Hearing Screening

**UNHS **– Universal Newborn Hearing Screening

**WHA **– World Health Assembly

## Competing interests

The author(s) declare that they have no competing interests.

## Authors' contributions

BOO designed the study. All authors contributed to data collection from their respective countries. BOO, DWS and BM drafted the manuscript. All other authors reviewed the draft and verified their respective country information. All authors read and approved the final manuscript. Contributors who did not participate in the manuscript preparation and did not meet the criteria for authorship have been listed in the acknowledgements.

## Pre-publication history

The pre-publication history for this paper can be accessed here:



## Supplementary Material

Additional File 1 Survey of infant hearing screening programmes in developing countries. Questionnaire completed by contributors to this project Click here for file
