# Supplementary material for: Progress towards early detection services for infants with hearing loss in developing countries
Source: BMC Health Serv Res. 2007 Jan 31;7:14. doi: 10.1186/1472-6963-7-14 (PMC1802737; doi:10.1186/1472-6963-7-14)
Supplement: Additional File 1 — Survey of infant hearing screening programmes in developing countries. Questionnaire completed by contributors to this project [file 1472-6963-7-14-S1.doc]

**SURVEY OF INFANT HEARING SCREENING PROGRAMMES**

**IN DEVELOPING COUNTRIES**

| NAME OF CONTRIBUTOR |  | | |
| --- | --- | --- | --- |
| E-MAIL |  | | |
| COUNTRY |  | NO. OF NHS SITES |  |

# Mark X to select

Kindly answer the following questions as accurately as you can:

| **1.** | **When and where was the first NHS programme implemented in your country?** | | | | | | | | | | | | | | | | | |  | |
| --- | --- | --- | --- | --- | --- | --- | --- | --- | --- | --- | --- | --- | --- | --- | --- | --- | --- | --- | --- | --- |
|  | Year |  | | Location: | | | | | | | | | | | | Urban | | |  | |
|  |  |  |  |  |  |  |  |  |  |  |  |  |  |  |  | Rural | | |  | |
|  | Type of Screening Site: | | | | | | | | | | | | | | | | | |  | |
|  | Government Hospital | | | | | | | | | | | | | | | | | |  | |
|  | Private Hospital | | | | | | | | | | | | | | | | | |  | |
|  | Community Health Centre | | | | | | | | | | | | | | | | | |  | |
| **2.** | **Which NHS models have been implemented in your country?** | | | | | | | | | | | | | | | | | | | |
|  | Targeted NHS: | | | | | | Hospital-Based | | |  | | | Community-Based | | | | | | |  |
|  | Universal NHS: | | | | | | Hospital-Based | | |  | | | Community-Based | | | | | | |  |
|  | Others (Please specify) | | | | | | | | | | | | | | | | | | | |
| **3.** | **What screening protocol is most widely used for your NHS programmes?** | | | | | | | | | | | | | | | | | | | |
|  | 1^st^ Stage | |  | | 2^nd^ Stage | |  | 3^rd^ Stage | | | |  | | | 4^th^ Stage | | | | |  |
|  | Risk Factors | |  | | Risk Factors | |  | TEOAE | | | |  | | | TEOAE | | | | |  |
|  | TEOAE | |  | | TEOAE | |  | DPOAE | | | |  | | | DPOAE | | | | |  |
|  | DPOAE | |  | | DPOAE | |  | AABR | | | |  | | | AABR | | | | |  |
|  | AABR | |  | | AABR | |  | ABR | | | |  | | | ABR | | | | |  |
| **4.** | **Is any of your screening programmes linked to any of the following WHO/UNICEF child health programmes?** | | | | | | | | | | | | | | | | | | | |
|  | Expanded Programme on Immunisation (EPI) | | | | | | | | | | | | | | | | | | |  |
|  | Baby-Friendly Hospital Initiative (BFHI) | | | | | | | | | | | | | | | | | | |  |
|  | Integrated Management of Childhood Illnesses (IMCI) | | | | | | | | | | | | | | | | | | |  |
| **5.** | **To what extent is Government involved in your NHS programmes?** | | | | | | | | | | | | | | | | | | | |
|  | Full Funding | | | | | | | | | | | | | | | | | | |  |
|  | Partial Funding | | | | | | | | | | | | | | | | | | |  |
|  | No Funding | | | | | | | | | | | | | | | | | | |  |
|  | If Partial Funding, what does it cover? | | | | | | | | | | | | | | | | | | |  |
|  | Equipment | | | | | | | | | | | | | | | | | | |  |
|  | Staffing | | | | | | | | | | | | | | | | | | |  |
|  | Office Space | | | | | | | | | | | | | | | | | | |  |
|  | Educational Materials | | | | | | | | | | | | | | | | | | |  |
|  | Others (please specify) | | | | | | | | | | | | | | | | | | | |
| **6.** | **Do patients pay for NHS services?** | | | | | | | | | | YES | | |  | | | | NO | |  |
|  | If yes, what services are paid for? | | | | | | | | | | | | | | | | | | |  |
|  | Screening Tests | | | | | | | | | | | | | | | | | | |  |
|  | Diagnostic Test | | | | | | | | | | | | | | | | | | |  |
|  | Provision of Hearing Aids | | | | | | | | | | | | | | | | | | |  |
| **7.** | **How will you rate patients’ attitude towards NHS programmes generally in your country?** | | | | | | | | | | | | | | | | | | | |
|  | Very Positive | | | | | | | | | | | | | | | | | | |  |
|  | Positive | | | | | | | | | | | | | | | | | | |  |
|  | Not Sure | | | | | | | | | | | | | | | | | | |  |
|  | Negative | | | | | | | | | | | | | | | | | | |  |
|  | Very Negative | | | | | | | | | | | | | | | | | | |  |
| **8.** | **How will you rate health workers (doctors and nurses) attitude towards NHS programmes generally in your country?** | | | | | | | | | | | | | | | | | | | |
|  | Very Positive | | | | | | | | | | | | | | | | | | |  |
|  | Positive | | | | | | | | | | | | | | | | | | |  |
|  | Not Sure | | | | | | | | | | | | | | | | | | |  |
|  | Negative | | | | | | | | | | | | | | | | | | |  |
|  | Very Negative | | | | | | | | | | | | | | | | | | |  |
| **9.** | **What would you consider to be the greatest achievement(s) and the greatest challenges of your NHS programmes?** | | | | | | | | | | | | | | | | | | | |
|  | Achievements: | | | 1. | | | | | | | | | | | | | | | | |
|  |  |  |  | 2. | | | | | | | | | | | | | | | | |
|  |  |  |  | 3. | | | | | | | | | | | | | | | | |
|  | Challenges: | | | 1. | | | | | | | | | | | | | | | | |
|  |  |  |  | 2. | | | | | | | | | | | | | | | | |
|  |  |  |  | 3. | | | | | | | | | | | | | | | | |
| **10.** | **In addition to NHS, what other routine hearing screening programmes exist for children in your country within the public healthcare or educational system?** | | | | | | | | | | | | | | | | | | | |
|  | Preschool | | |  | | School Entry | | |  | | | None | | | | |  | | | |
| General Comments or Suggestions | | | | | | | | | | | | | | | | | | | | |
